# Human foodborne listeriosis in England and Wales, 1981 to 2015

**DOI:** 10.1017/S0950268820000473

**Published:** 2020-02-19

**Authors:** J. McLauchlin, K. A. Grant, C. F. L. Amar

**Affiliations:** 1 Public Health England Food Water and Environmental Microbiology Services, National Infection Service, Colindale, London. NW9 5EQ, UK; 2 Public Health EnglandGastrointestinal Bacteria Reference Unit, National Infection Service, 61 Colindale Avenue, London. NW9 5EQ, UK

**Keywords:** Foodborne illness, *Listeria monocytogenes*, listeriosis, outbreaks, sporadic cases, surveillance

## Abstract

Almost all cases of human listeriosis are foodborne, however the proportion where specific exposures are identified is small. Between 1981 and 2015, 5252 human listeriosis cases were reported in England and Wales. The purpose of this study was to summarise data where consumption of specific foods was identified with transmission and these comprised 11 sporadic cases and 17 outbreaks. There was a single outbreak in the community of 378 cases (7% of the total) which was associated with pâté consumption and 112 cases (2% of the total) attributed to specific foods in all the other incidents. The proportion of food-attributed cases increased during this study with improvements in typing methods for *Listeria monocytogenes*. Ten incidents (one sporadic case and nine outbreaks of 2–9 cases over 4 days to 32 months) occurred in hospitals: all were associated with the consumption of pre-prepared sandwiches. The 18 community incidents comprised eight outbreaks (seven of between 3 and 17 cases) and 10 sporadic cases: food of animal origin was implicated in 16 of the incidents (sliced or potted meats, pork pies, pâté, liver, chicken, crab-meat, butter and soft cheese) and food of non-animal origin in the remaining two (olives and vegetable rennet).

## Introduction

Listeriosis is predominantly a foodborne illness caused by the bacterium *Listeria monocytogenes* which is recognised as a major foodborne pathogen and is the most common cause of death from foodborne illness in the European Union [1]. The disease is most likely to present as a severe systemic infection: affected patient groups include, those over 60 years of age, the immunocompromised as well as the pregnant women and their unborn or newborn infants [2]. Foods associated with listeriosis can be contaminated at primary production, or more frequently, become contaminated from food production environments where the bacterium colonises harbourage sites for years and even decades [3]. Following consumption of contaminated food, the disease has a low attack rate and a variable (1–90 days) incubation period [4, 5]. Cases occur both sporadically or as outbreaks. Because of national and international food distribution chains, cases in outbreaks related to common food exposures can be both temporally and geographically widely distributed. Consequently, there are difficulties in linking specific foods to infection. Furthermore, since many of the clusters identified are small (<5 cases), analytical epidemiological studies are unlikely to be effective and investigation is reliant on a combination of other approaches. Considerable information has been generated from the investigation of listeriosis outbreaks since the 1960s [2, 6]. However, because of the difficulties outlined above in investigating human listeriosis and identifying the appropriate interventions in the food chain, the proportion of all the cases where a specific exposure is identified is very small. Hence it is important to consolidate data from both outbreaks and sporadic cases to provide a better understanding of control of this disease.

In England and Wales, human listeriosis has been investigated since the 1980s and both foodborne outbreaks and single sporadic cases associated with the consumption of a range of foods were identified [7–20]. However, less than half of the foodborne incidents identified have been described in the peer reviewed literature. In December 2015, whole genome sequencing (WGS) was introduced by the Public Health England's (PHE) national reference laboratory as a routine method for the surveillance of human listeriosis in England and Wales, which has been successfully used for cluster and outbreak detection, and the characterisation of *L. monocytogenes* isolates from food and the environment. The purpose of this study was to review foodborne listeriosis in England and Wales between 1981 and 2015 prior to the introduction of WGS. Available epidemiological and microbiological data from each incident (including information not previously described) were reviewed together with evidence for exposures linked to foodborne transmission, and information which initially identified the implicated food vehicles.

## Materials and methods

### Case definitions, strength of evidence for foodborne transmission

Data on cases of human listeriosis in England and Wales between 1981 and 2015 were considered. A case of listeriosis was defined as a person with an illness clinically compatible with a diagnosis of listeriosis with the isolation of *L. monocytogenes,* usually from a normally sterile anatomical site. For pregnancy associated cases, a mother and her unborn or newly delivered infant(s) were considered as a single case. Voluntary reporting of cases to a central database occurred until 2010 when the PHE, The Health Protection (Notification) Regulations 2010 (http://www.legislation.gov.uk/uksi/2010/659/pdfs/uksi_20100659_en.pdf), made reporting of all human listeriosis cases mandatory. Only cases where isolates were sent for characterisation to the national reference laboratory (Gastrointestinal Bacteria Reference Unit, GBRU) are considered here.

For this study, the nature and strength of the evidence linking consumption of a particular food to incidents of foodborne listeriosis (sporadic cases or outbreaks) was based on the general reporting criteria proposed by the European Food Safety Authority for outbreaks due to any agent [21]. Strong epidemiological evidence was defined as either that with a statistically significant association with consumption of a specific food from an analytical study, or convincing descriptive evidence of specific food consumption. Strong microbiological evidence constituted the identification of an indistinguishable *L. monocytogenes* isolate from a case or cases as well as from food, a food component or its environment which is unlikely to have been contaminated either coincidentally or after the event. Strong evidence was also provided by comprehensive product-tracing investigations where a place or common point of exposure (including a point of sale) was identified along the food-production and distribution chain for the case or a large proportion of cases.

### Data sources

Cases of listeriosis diagnosed by microbiology laboratories isolating the bacterium from clinical specimens (most often blood or cerebrospinal fluid) were reported by microbiology staff either via paper reports or through electronic data capture. Isolates from clinical laboratories were referred to the national *Listeria* reference laboratory located in the PHE Gastrointestinal Bacteria Reference Unit in London.

Brief clinical details of each case were collected by the hospital staff using a structured questionnaire (https://www.gov.uk/government/publications/listeria-enhanced-surveillance-questionnaire-for-microbiologists). Where patients were still in the hospital, food preference questionnaires were administered to patients by clinical staff, about food exposures prior to the onset of illness (https://www.gov.uk/government/publications/listeria-enhanced-surveillance-questionnaire). Where cases returned to the community, the food preference questionnaires were administered to patients or an immediate family member (if the patient was either too infirm or had died) by local Environmental Health Practitioners (EHPs) or staff within Health Protection Teams.

### Data from testing of foods and the environment

Foods or environmental swabs were collected by EHPs from a range of settings including patient's homes, at retail or other settings common to patients, or from the point of manufacture. Sample collection was performed for various purposes including as part of outbreak or incident investigations, in response of unrelated complaints by members of the public, as part of ‘routine’ inspection of food businesses or as part of local or national coordinated microbiology surveys.

Food and environmental samples were collected and transported in accordance with standard codes of practice [22] and were microbiologically tested by Official Control Laboratories located throughout England and Wales using standard methods. The current methods applied for the presence and enumeration of *Listeria* spp, including *L. monocytogenes* was performed according to the ISO 11290 methods [23, 24].

### Data from characterisation of *L. monocytogenes* cultures

Confirmation of identity of *L. monocytogenes* was initially performed by clinical or food water and environmental microbiology laboratories using standard methods and isolates (together with associated meta-data) were referred to the PHE Gastrointestinal Bacteria Reference Unit, London for confirmation and strain characterisation. The submission of *L. monocytogenes* cultures was voluntary throughout. The methods used by the reference unit for species confirmation varied over the study period. Initially biochemical tests were used and these were superseded by molecular tests. There was variation between the methods used in the reference laboratory with those in the clinical or Official Control laboratories testing food. Cases were only included in this series where a *L. monocytogenes* from a clinical specimen was confirmed by the reference laboratory.

A range of techniques was used to subtype *L. monocytogenes* isolates including serotyping by agglutination using anti-sera [25], gel-based PCR serogrouping [26] and real-time PCR serogrouping [27]. Discriminatory sub-typing methods were applied based on: phage-typing [28], amplified fragment length polymorphism (AFLP; [29]), pulsed-field gel electrophoresis [30] and fluorescent AFLP (fAFLP) [31]. The time periods when each of these techniques were used is shown in Table 1. Selected isolates were also retrospectively tested by WGS from 2012 to 2015 [19, 32] providing clonal complex (CC) grouping in accordance with the Institut Pasteur international multi-locus sequence type (MLST) database. (http://bigsdb.pasteur.fr/listeria/listeria.html) as well as high resolution characterisation and phylogenetic information by single nucleotide polymorphism analysis [33].

**Table 1.** Total numbers of reported listeriosis cases, outbreaks and sporadic cases linked to specific foods: England and Wales 1981–2015

Time period	1981–1986	1987–1989	1990–2001	2002–2006	2007–2015
Total numbers of cases reported	604	753	1353	957	1585
Mean annual totals (range of cases reported per year)	101 (58–136)	251 (237–278)	113 (90–146)	191 (139–233)	176 (147–226)
Incidents (cases) linked to specific foods					
Numbers of incidents in hospitals (cases)	0	0	1 (4)	4 (10)	5 (23)
Numbers of outbreaks in the community (cases)	0	1 (378)	0	1 (17)	6 (48)
Numbers of sporadic cases linked to specific foods	1	3	0	2	4
% of cases linked to specific foods	0.2%	51%^a^	0.3%	3%	5%
Methods used for characterisation of *L. monocytogenes*	ST, PT	ST, PT	ST, PT	ST, PT, AFLP, PFGE	MST, fAFLP, WGS

Characterisation methods used were: ST, serotyping; PT, phage-typing; AFLP, amplified fragment length polymorphism; PFGE, pulsed-field gel electrophoresis; MST, molecular serotyping; fAFLP, fluorescent amplified fragment length polymorphism; WGS, whole genome sequencing (selected isolates only).

a If the outbreak associated with pâté (O1) is excluded from this period, 0.8% of the total cases were linked to specific foods.

### Data collation and incident identification

A national register for all cases of listeriosis in England and Wales was maintained by PHE Gastrointestinal Bacterial Reference Unit in London. This national database combines clinical and epidemiological data with strain typing data on *L. monocytogenes* from patient's specimens. The national case register was periodically interrogated to identify both clusters of cases infected by indistinguishable *L. monocytogenes* cultures, as well as with matching isolates from food(s) and the environment with those from human cases. The available data from the food history questionnaires was also reviewed both nationally and locally with the aim of identifying any common risk factors or exposures and to confirm the consumption of any implicated food item.

### Basic data sets collected for this study

**Fig. 1.**
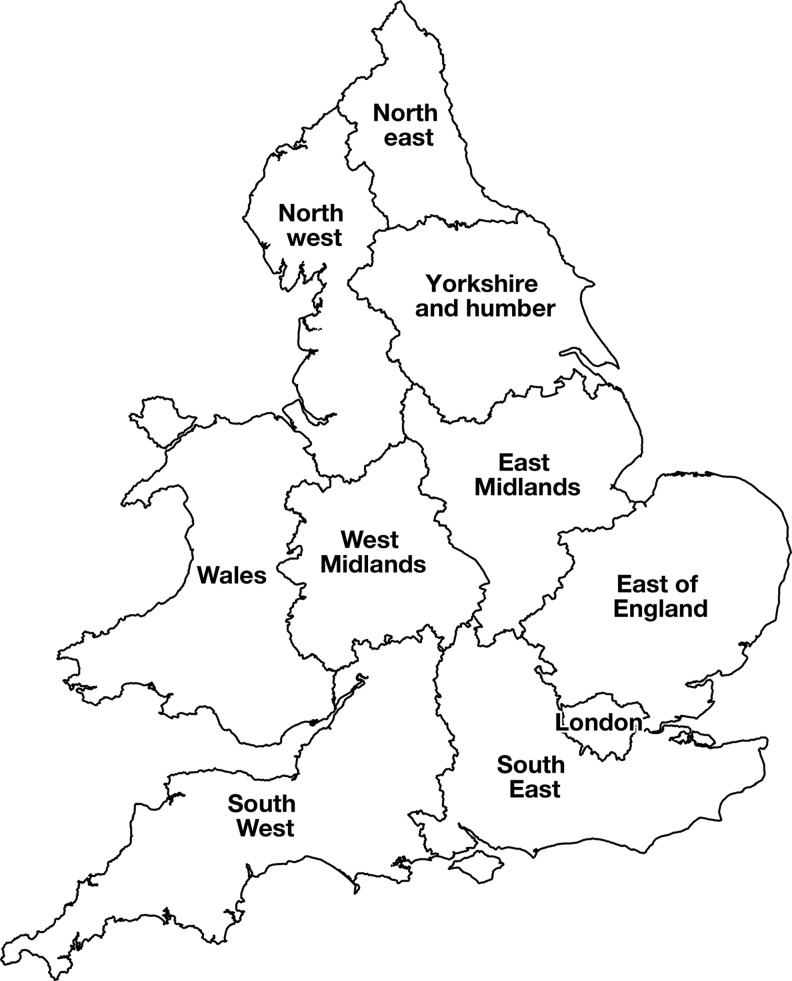
Regions of England and Wales used for this study.

For the purpose of this review, a basic dataset was collected for all incidents (including those already published in the peer reviewed literature) which were reported to PHE (or its predecessor organisations) in England and Wales between 1981 and 2015. Due to the time period covered, complete data were not always available. The basic dataset included: numbers and characteristics of the case(s); location of home address in terms of Regional Health Authorities regions (Fig. 1); the month and year of onset (or date of the first specimen where the bacterium was recovered); the settings within the food chain where *L. monocytogenes* was recovered and where available, levels of the bacterium detected in food samples. Data on the *L. monocytogenes* characterisation methods used as well as the strength of analytical or descriptive epidemiological analysis of any associations with food were also documented. Finally, information was also considered on the initial observation that provided evidence to link a specific food to disease transmission.

## Results and discussion

### Human listeriosis in England and Wales, 1981–2015

The aim of this report was to review data on the investigations of foodborne listeriosis in England and Wales between 1981 and 2015 which includes reviewing data in the peer reviewed literature together with hitherto unpublished data. Between 1981 and 2015, a total of 5252 cases of human listeriosis in England and Wales were reported. This report highlights the importance of submission of *L. monocytogenes* isolates from clinical cases of listeriosis for characterisation which is essential to establish foodborne links. We estimate that isolates are received by the national reference laboratory from >95% of cases reported (PHE unpublished data).

Listeriosis cases were considered over the five time periods: 1981–1986, 1987–1989, 1990–2001, 2002–2006 and 2007–2015 (Table 1). Prior to 1987, there were between 58 and 136 cases reported per year and from 1987–1989 this increased to 237–278 cases per year (Table 1) with just over half the number of cases associated with a large outbreak (outbreak O1 [11]). There then followed a decline in reported cases between 1990–2001, with annual totals ranging from 90 to 146 cases. The number of cases per year increased to between 139 and 233 between 2002 and 2015 (Table 1).

Amongst all the 5252 cases, there were 28 incidents (480 cases) where consumption of specific foods was identified as associated with transmission of listeriosis, and these comprised 9% of the total reported cases. The 28 incidents included 11 sporadic cases and 17 outbreaks. Ten incidents occurred in hospitals (nine outbreaks and one sporadic case, total 37 cases) and the remaining 18 incidents (10 sporadic cases and eight outbreaks, total 443 cases) occurred in the community. The large outbreak occurring between 1987 and 1989 resulted in 51% of reported cases attributed to a specific food during this period. However, if the cases from this outbreak are excluded, 0.8% of all the cases reported between 1987 and 1989 were identified as linked to specific foods. Therefore, between 1981 and 2001 and excluding the large outbreak, ⩽0.8% of cases were linked to the consumption of specific foods. The proportion of the total numbers of cases linked to specific foods increased to 3% between 2002 and 2006 with the implementation of AFLP and then to 5% between 2007 and 2015 with fAFLP being the primary discriminatory typing tool (Table 1).

During the 1981 to 2015 study period, a variety of techniques were used to characterise *L. monocytogenes*, and these techniques provided sequential improvements in reproducibility, discrimination and typability. With these improvements in the characterisation of this bacterium, there was increased attribution of cases to specific foods. Despite the efforts to investigate cases, it was not until the advent of more robust and discriminatory typing systems for *L. monocytogenes* (particularly AFLP and fAFLP) that the proportion of cases attributed to specific foods associated with transmission increased to over 1%: this highlights the need to apply optimal typing techniques not only to isolates from clinical cases, but also to those from food (see below).

In December 2015, PHE introduced WGS for routine analysis of all isolates submitted to the reference laboratory. At the time of writing in 2019, out of approximately 500 listeriosis cases, WGS increased the percentage of cases linked to specific foods to ~10% (PHE, unpublished data).

This review is timely since the improvement in typing from WGS will further continue to increase the identification of similarities between clinical and food/environmental isolates within England and Wales, and this is likely to be enhanced as other countries adopt the use of WGS. The possibilities of international linkage were illustrated by the outbreak of listeriosis occurring between 2015 and 2018 and associated with frozen sweetcorn produced in Hungary [34]. Related cases were identified by the use of WGS not only in the UK, but also in Austria, Australia, Denmark, Finland, Hungary and Sweden. However, the utilisation of this advance in technology by analysis of WGS data will only provide maximal public health benefits to surveillance when combined with other data inputs, hence the emphasis in this review to re-examine data from historic incidents.

### Incidents in hospitals

The 10 incidents occurring in hospitals (H1–H10; Table 2), comprised a single sporadic case (H5), and nine outbreaks involving between two and nine cases (Table 2). Seven of these outbreaks have previously been described in the literature [12, 13, 15–17]. All 10 incidents were associated with the consumption of pre-prepared sandwiches served in this setting: one of the outbreaks also implicated a salad product (H8, [16]).

**Table 2.** Characteristics of listeriosis in hospitals associated with pre-prepared sandwich consumption: characteristics of nine outbreaks and a sporadic case which occurred in England and Wales, 1981–2015

Outbreak/incident number	Year	Number of cases	Regions	References
H1	1999	4	NE	[12]
H2	2003	2	W	[15]
H3	2003	5	SW	[13]
H4	2004	2	E	[17]
H5	2007	1	L	(PHE unpublished)
H6	2008	3	NW	[17]
H7	2010–13	9	NE	[17, and PHE unpublished]
H8	2011	3	WM	[16]
H9	2013–14	4	NW, NE	(PHE unpublished)
H10	2014	4	SE	(PHE unpublished)

Regions: L, London; E, East; NW, North West; WM, West Midlands; NE, North East; SE, South East; W, Wales

The duration of all outbreaks ranged from 4 days (H2) to 32 months (H7). All cases occurred within immunocompromised adults who were >60 years of age except for outbreak H3 which affected five pregnant women and their unborn infants who ate sandwiches from a retail outlet in hospital whilst attending antenatal clinics [13]. Eight of the nine outbreaks occurred within single regions: three (H1, H6 and H9) in more than one hospital within the same regions. The final outbreak (H9) occurred in hospitals across two adjacent regions and involved four cases.

For all 10 incidents, there was descriptive epidemiological evidence linking the cases to sandwich consumption: in one outbreak, a salad product was also implicated (H8, [16]). In nine of these incidents, *L. monocytogenes* of the same type was recovered from patients' specimens as well as from the implicated sandwiches collected either within the hospital, and or from the point of manufacture (sandwiches or environmental sites) and therefore were categorised as having both strong microbiological and epidemiological evidence for the consumption of this specific food. For the 10th incident (H8), there was strong descriptive epidemiological evidence for sandwiches and a salad product but the implicated type of *L. monocytogenes* was not isolated from these foods [16]. However, in this incident, all three cases were in the same hospital with a common exposure to sandwiches and salad from a single manufacturer: strong evidence was provided by product-tracing investigation based on patient menu choice records and food supplier records.

There was no information on levels of *L. monocytogenes* in sandwiches or fillings in one of the outbreaks (H1). The bacterium was detected in all samples at less than 10^2^ cfu/g in products collected at various points in the food chain, including in finished sandwiches collected in hospitals ready for consumption in seven of the incidents (H2, H3, H6, H7, H8, H9 and H10). In the final two incidents (H4 and H5), *L. monocytogenes* was detected at >10^2^ cfu/g in complete sandwiches and fillings collected at the point of manufacture.

For one of the incidents (H8) there was no information on the ingredients used in the sandwiches, but for the remaining, sandwiches with various types of fillings were contaminated with the implicated strains: seven contained various cooked meats, two eggs, five cheese, six salad or other plant based materials and five with fish or crustaceans. In eight of the incidents, the implicated strain was recovered from environmental sites, utensils or equipment within the sandwich production environments and, as previously identified [17], provided evidence for cross-contamination at these factories. Although contamination of an ingredient or ingredients from suppliers to the sandwich manufacturers cannot always be excluded, the most likely reservoirs of contamination were from sites within the sandwich production environments. There was evidence of poor temperature control (>8 °C) of the sandwiches at the hospitals in five of the incidents (H2, H4, H5, H7 and H8).

In six of these nosocomial outbreaks, local, or (in H3) national increases of cases in one or more hospitals were detected and, following patient interviews or review of hospital food consumption records, preliminary information linked these clusters to pre-prepared sandwich consumption (Table 3). For three of the outbreaks (H5, H9 and H10), the source of infection was indicated by reviewing *L. monocytogenes* typing data of isolates obtained from unrelated microbiological testing. For the single sporadic case (H5), unrelated testing identified high (>10^2^ cfu/g) levels of *L. monocytogenes* in pre-packed sandwiches which were notified by the producer and this led to a review and identification of a case infected with the same strain in a hospital supplied by the producer.

**Table 3.** Initial observations that provided evidence to link outbreaks and incident of foodborne listeriosis to specific foods

Initial observations	Nosocomial incidents and outbreaks	Community incidents and outbreaks
Local and or national surveillance and recognition of increase in cases.	H1, H2, H3, H4, H6, H7, H8	
Unrelated food testing and, following submission of *L. monocytogenes* to the reference laboratory for characterisation, the recognition of the same strain amongst isolates from clinical cases of listeriosis.	H5, H9, H10	O1, O2, O4, O6, O7, O8, S5, S9
Food testing of contents of domestic or institutional refrigerator and isolation of the same *L. monocytogenes* strain as recovered from clinical cases of listeriosis.		O3, S1, S3, S4, S7, S8
Patient suspicion of implicated food or retailer.		O5, S2, S6, S10

As previously reported [17], the most common food type associated with transmission of listeriosis in England and Wales during 1981 to 2015 was pre-prepared sandwiches served in hospitals. The duration of these nosocomial outbreaks ranged from a few days to 32 months and two thirds occurred within a single hospital. Of the three outbreaks that occurred in more than one hospital, the affected patients were in hospitals within the same or in adjacent regions which were supplied by the same manufacturer and this reflects distribution patterns for this short shelf life (usually <5 days) food product. At the time of writing (2019), similar incidents with pre-prepared sandwiches served in hospitals have continued to occur [35]. Although all the incidents in England and Wales described here were either sporadic or small outbreaks generally affecting less than five cases, there is the potential to affect greater numbers of patients, and the incidents described here show many similarities to an outbreak that occurred in Canada in 2008 where 57 cases and 24 deaths occurred [36].

It is intriguing that the 10 incidents that occurred in hospitals all had an association with pre-prepared sandwiches, and that this food type was not recognised in any of the outbreaks or sporadic cases or in the community (see later text). Although foods associated with transmission are only identified in a small proportion of cases overall, this may reflect differences in testing between products served in hospitals and those on retail sale. However, it may be important that the microbiological testing of sandwiches in hospitals detected *L. monocytogenes* at <10^2^ cfu/g in the majority of instances: higher levels (>10^2^ cfu/g) of contamination were detected in almost all listeriosis incidents in the community (see later text). Sandwiches are complex foods with multiple components with differing durability parameters. Although components within sandwiches are likely to support the growth of the bacterium, the shorter shelf life as compared to some of the other foods associated with the community incidents is consistent with exposure to *L. monocytogenes* at low levels being of greater risk for infection to immunocompromised patients in hospitals than the general population in the community. Evidence from the incidents described here indicates there are opportunities for improvements in temperature control at hospitals. This may be difficult in these types of environment and the most effective interventions (as with the incidents in the community) are to eliminate and reduce contamination at the point of production.

### Incidents in the community

There were 18 incidents in the community; eight outbreaks (Table 4) and 10 sporadic cases (Table 5).

**Table 4.** Characteristics of foodborne listeriosis outbreaks in the community: England and Wales 1981–2015

Outbreak Number	Month and year of onset	Number of cases	Regions	Food type	Epidemiological evidence	Clinical microbiological evidence	Food microbiological evidence	References
O1.	January 1987–July 1989	378 (more than 50% associated with pregnancy)	All^a^	Pâté	Case/case study showed a significant associated between the consumption of pâté and infection with the types recovered from the pâté produced from a single manufacturer as compared to cases infected by other types	All patients infected by one of two *L. monocytogenes* strains (serovar 4b) and indistinguishable by phage typing)	Nationwide survey in July 1989 of pâté on retail sale showed that 48% of samples from a single Belgian manufacturer were contaminated with *L. monocytogenes* (11% at >10^3^ cfu/g) and 96% were indistinguishable from those isolated from the cases. Samples from this manufacturer were more likely to be contaminated than those from other manufacturers.	[11, 37, 38]
O2.	Jan–July 2003	17 (11 cases pregnancy associated, six cases >50 years of age, four were immunocompromised)	Y&H, NE	Butter	Cases and controls were interviewed and exposure to butter in 2 kg catering packs from the implicated dairy and used by local sandwich outlets rather than butter used in the home was identified as a significant risk factor.	*Same L. monocytogenes* strain (serovar 4b AFLP type V and indistinguishable by phage-typing) isolated from blood (14 cases), CSF (two cases) and both blood and CSF (one case).	Routine testing in April 2003 identified *L. monocytogenes* contamination of butter produced at a dairy in the Yorkshire and Humberside region. *L. monocytogenes* indistinguishable from that recovered from the cases were recovered from seven different batches of butter at levels of between <20 to 1.8 × 10^2^ cfu/g. In four subsequent batches of butter from the same dairy in April and May, the same type of *L. monocytogenes* was detected at <20 cfu/g. The same *L. monocytogenes* strain was also isolated from drains within the factory in June and July 2003 but not from butter.	[14, PHE unpublished]
O3.	July–November 2009	14 (one pregnancy associated case, 13 non-pregnant, 10 >60 years of age and 11 immunocompromised)	Y&H, SE, SW, WM, NW, W^b^	Sliced cooked meats	Food histories from all nine patients in this outbreak were 9 times more likely to report consumption of sliced meat than patients infected by other types, and were 23 times more likely to shop in smaller types of shops	All patients infected by the same *L. monocytogenes* strain (serogroup 4 and fAFLP type IV4.3) which was isolated from blood (11 patients), CSF (one patient) or ascitic fluid (one patient): the source of the isolate from the final patient was not recorded.	The *L. monocytogenes* strain recovered from the patients was isolated from environmental sites and food samples from a manufacturer of cooked and sliced meats in the North West in March and October 2009. The sliced meat producer had a complex onward distribution chain to retailers as well as wholesalers for further national distribution. The *L. monocytogenes* strain had been recovered between July and August 2009 at levels of between 20 and 10^3^ cfu/g from sliced turkey products and ⩽10 cfu/g for other meat products, including those used in sandwiches. One of the patients (a 92-year-old female) reported consuming sliced meat from a local bakery in November 2009 and products from the implicated manufacturer yielded the outbreak strain of *L. monocytogenes* from sliced beef and sliced turkey at between ⩽10 and 10^4^ cfu/g.	(PHE Unpublished)
O4.	October 2009-November 2010	10 (all non- pregnant and aged 48–81 years)	L, Y&H, E, NW, WM	Sliced cooked meats	All cases reported histories of sliced cooked meat consumption with seven reporting eating sliced ham and three tongue.	The same *L. monocytogenes* strain (serovar 1/2a, fAFLP XIV.6a) was recovered from blood (eight cases), CSF (one case) and blood and CSF (one case).	The same strain of *L. monocytogenes* was recovered at <20 cfu/g from sliced meat products (ham, corned beef and beef or ham sandwiches) collected at retail between August and October 2009. The products originated from a single manufacturer in the Yorkshire and Humberside Region which supplied small retailers and convenience stores with sliced meats, pre-prepared sandwiches and wraps. In February 2010, this manufacturer recalled sliced cooked chicken roll products due to *L. monocytogenes* contamination. Further testing of samples collected between February and November 2010 recovered the same strain of *L. monocytogenes* from 13 different retail stores in the East of England, Yorkshire and Humberside and Wales as well as product collected at the point of manufacture. All the products (sliced pork, corned beef, pork luncheon meat, chicken rolls and ham sandwiches) originated from this manufacturer. *L. monocytogenes* was detected in all samples at <20 cfu/g except for samples tested in March 2010 (two chicken rolls contaminated at 10^3^ cfu/g and sliced pork at 10^2^ cfu/g) and a single sample of luncheon meat (10^2^ cfu/g) in June 2010.	(PHE Unpublished)
O5.	May 2010–July 2012	13 (all age >50 years)	Y&H, E^c^	Pork pies	Index patient associated infection with pork pie consumption. Nine of the remaining patients reported eating pork pies. Amongst all listeriosis cases in England during 2010 to 2012, those that consumed pork pies were significantly more likely to be infected with the outbreak strain and reside in the Yorkshire and Humber region than listeriosis patients infected by other strains	All patients infected by the same *L. monocytogenes* strain (serogroup 4, fAFLP I.74/I.799 MLST CC2) was isolated from blood (nine patients) or CSF (four patients)	The *L. monocytogenes* outbreak strain was recovered at <20 cfu/g from four out of seven pork pies collected from seven retailers in South Yorkshire or the East Midlands, all produced by the same manufacturer in the Yorkshire and Humber Region in 2012. The outbreak strain was also recovered from finished product (<20 cfu/g) and environmental sites within the manufacturing environment. The outbreak strain was detected at 10^4^ cfu/g in a single pork pie from the point of sale (a market stall identified by the patient): there was no information available on storage and condition of the pork pie after purchase and before submission to the laboratory for testing.	[19]
O6.	October 2011–April 2013	3 (all aged >55 years and with underlying illnesses)	L	Crab meat	Two cases in 2012 and one in 2013. Two of the cases reported either consumption of fresh crab or dressed-crab purchased in London from a fish market and from a fishmonger: the third case reported consumption of other types of seafood but did not specifically identify consuming crab-meat.	The same *L. monocytogenes* strain (serogroup 4, fAFLP type I.72, CC22) was isolated from blood (two cases) or CSF (one case).	*The same L. monocytogenes* strain was isolated from dressed-crab and environmental samples from a producer in the North East of England in September 2010 and in November and December 2012. One of the food samples from 2012 contained levels of *L. monocytogenes at* >10^2^ cfu/g. Further sampling was carried out at the factory and retail outlets in London in May 2013. The same *L. monocytogenes* was isolated from food and environmental samples from the crabmeat producer and from dressed crab sampled at the end of shelf life at >10^2^ cfu/g.	[20]
O7.	July 2012–September 2013	5 (all patients aged >60 years)	NW	Cooked pressed beef set in gelatin	Three patients had a history of consuming pressed beef purchased from different retailers and made by a single producer. The fourth case purchased raw meat from a butcher's shop also supplied with cooked meat products from the same producer.	The same *L. monocytogenes* (serovar 1/2a, fAFLP type XI.23, CC155) was isolated from all patients' blood	Prior to recognition of cases, the same strain of *L. monocytogenes* was recovered from pressed beef from a single manufacture at <20 cfu/g at retail in the North West in February and April 2012. Further food samples were collected between September and December 2012 from two butchers, two market stalls, three other retailers where cases purchased pressed beef. The same *L. monocytogenes* strain was isolated from all premises at <20 – 10^2^ cfu/g. *L. monocytogenes* was not recovered from products collected at the manufacturer in 2012, although this strain was recovered from environmental sites and food from the producer in April 2013.	(PHE Unpublished)
O8.	February–August 2013	3 (one case pregnancy associated two aged >80 years)	WM, Y&H	Crab meat	All three patients reported eating crab meat and shellfish prior to onset of symptoms.	All patients infected by the same *L. monocytogenes* strain (serogroup 4 fAFLP type V.3 type, CC1) which was isolated from all patients' blood.	The *L. monocytogenes* strain was isolated in September 2011 from crab meat sampled from a single producer in the North East. The producer sold cooked crab meat through mobile vendors throughout England. In November to December 2011 the same strain of *L. monocytogenes* was recovered from 21 samples of final product at 10^5^ cfu (one sample), 20–40 cfu/g (three samples) and <20 cfu/g (17 samples). In April 2012 *L. monocytogenes* was recovered from three final products collected at the point of production, two at 10^4^ cfu/g and one at <20 cfu/g. After the recognition of an association with human cases of listeriosis, further sampling was carried out between September and December 2013 and the same *L. monocytogenes* strain was recovered from six out of 82 environmental swabs and 100 (40%) of 247 samples of final product taken from the factory environment: 16% of the final product samples contained *L. monocytogenes* at >10^3^ cfu/g, 19% at 10^2^–10^3^ cfu/g, 24% at 20 to <10^2^ and 41% at <20 cfu/g.	[20]

L, London Region; E, East Region; NW, North West Region; Y&H, Yorkshire and Humberside Region; WM, West Midlands Region; NE, North East Region. CSF, cerebrospinal fluid; AFLP, amplified fragment polymorphism; fAFLP, fluorescent amplified fragment polymorphism; cfu, colony- forming units.

a Cases also occurred in Scotland and Norther Ireland.

b One case in Scotland.

c Index case resident in the South East but purchased food from the East Midlands region.

**Table 5.** Characteristics of sporadic foodborne listeriosis cases in the community: England and Wales 1981–2015

Case Number	Month and year of onset	Regions	Food type	Epidemiological evidence	Clinical microbiological evidence	Food microbiological evidence	References
S1	January 1986	L	Soft cheese	Patient interview identified consuming soft cheese	*L. monocytogenes* serovar 4b isolated from the CSF of a 36 year-old non-pregnant female	*L. monocytogenes* indistinguishable by phage typing isolated from remnant of a French cheese collected from the patient's domestic refrigerator	[7]
S2	February 1988	L	Soft cheese	Interview with patient's family identified consuming soft cheese and retailer where this was purchased	*L. monocytogenes* serovar 4b was isolated from the CSF of a 40 year-old non-pregnant female	*L. monocytogenes* indistinguishable by phage typing heavily contaminated unopened English soft cheeses collected at retail in February 1988. During the rest of 1988, the same *L. monocytogenes* strain was isolated from 16 of 24 cheeses sampled at retail and 12 out of 24 cheeses and shelving sampled from the factory which was located in the South of England. *L. monocytogenes* sampled from cheeses at the factory was present at <10 cfu/g, but 6 of 16 samples tested at retail were contaminated at between 10^5^ and 10^7^ cfu/g	[9, 10]
S3	1988	Y&H	Cooked chicken	Patient interview identified consuming cooked chicken	*L. monocytogenes* (serogroup 4) was recovered from maternal blood and sites on a spontaneously delivered non-viable 23 week foetus	*L. monocytogenes* indistinguishable by phage typing was recovered from remnants of a commercially prepared cooked chicken from the patient's domestic refrigerator	[8]
S4	1988	Y&H	Vegetable rennet	Patient interview identified consuming vegetable rennet	*L. monocytogenes* serogroup 4 was isolated from placental swabs, maternal blood and foetal blood from a 23 week miscarriage	*L. monocytogenes* indistinguishable by phage typing was recovered from a 3-month-old bottle of vegetable rennet collected from the patient's domestic refrigerator	[8]
S5	July 2005	NW	Sliced cooked meat	The patient reported eating cheese and sliced ox tongue purchased from a local supermarket.	*L. monocytogenes* serovar 1/2b (AFLP II) was isolated from the blood of an 84 year-old female	*L. monocytogenes* indistinguishable by AFLP and PFGE was isolates recovered by unrelated testing of sliced meat	(PHE unpublished)
S6	September 2006	L	Sliced cooked meat	Patient reported eating sliced meats from a single producer	*L. monocytogenes* serotype 1/2a AFLP type VII was recovered from the blood of a 72 year-old female	The same strain by AFLP and PFGE was detected in sliced honey roast ham sampled from the producer in December 2006. Over the following 3 months, a further 55 *L. monocytogenes* isolates were recovered from samples collected from this producer: 10 from meat slicing machines and environmental samples, and the remaining 45 were from sliced ham at <20 to 10^3^ cfu/g. Five different *L. monocytogenes* strains were recovered from these samples, none of which was the same strain as that recovered from the patient.	(PHE unpublished)
S7	May 2008	L	Chopped cooked liver	Nursing home staff identified patient had consumed cooked liver	*L. monocytogenes* serotype 1/2a, fAFLP type III.10 was isolated from the blood an 89 year-old female	Samples of food were collected from a refrigerator in the nursing home and the same strain of *L. monocytogenes* as recovered in cooked chopped liver at 10^2^−10^3^ cfu/g. The cooked liver was prepared by a local delicatessen and the same *L. monocytogenes* strain was detected at <20 cfu/g from this product as well as from a conveyer belt, a meat slicer and a preparation sink.	(PHE unpublished)
S8	July 2009	E	Olives	The patient identified consuming olives in garlic and sweet chili oil	*L. monocytogenes* serogroup 4, fAFLP type UD4.1 was isolated from the blood of 29 year-old female who delivered an infant with listeriosis at 39 weeks by caesarean section.	The patient was interviewed and olives in garlic and sweet chili oil were recovered from the patient's refrigerator and yielded the same *L. monocytogenes* type (serotype 4, fAFLP type UD4.1) at 40 cfu/g. Olives and environmental samples were taken from the local market stall identified by the patient as the place of purchase as well as the local food business operator who marinated the olives and supplied these to the market stall-holder. The same type of *L. monocytogenes* as recovered from the patient was also recovered from black olives in garlic and sweet chili sauce, a wooden serving bowl from the market stall and a cutting blade used to chop garlic for the marinade at the point of production. Except for the sample collected from the patient's home, *L. monocytogenes* was recovered from olives at <20 cfu/g.	(PHE unpublished)
S9	October 2011	NW	Sliced ox tongue	Patient identified eating sliced meats from a local retailer	*L. monocytogenes* serovar 1/2b, fAFLP type IVb19, CC59 was isolated from the blood of a 70 year-old male	The same strain was recovered at <10 to 120 CFU/g from cooked ox tongue in April 2011 at retailer and at the producer in July 2011. Further isolates of this strain were obtained from cooked ox tongue collected at retail which were obtained in April and October 2011 at levels of between 50 and 10^4^ cfu/g in opened and unopened packs including from the local retailer identified by the patient.	[18]
S10	June 2012	NW	Sliced cook meat	Patient identified consuming ox-tongue from a local supermarket	*L. monocytogenes* serogroup 1/2a (fAFLP type IX.14) was isolated from the blood of a 72 year-old female	Sampling at the supermarket identified by the patient as the place of purchase recovered 18 *L. monocytogenes* isolates of the same fAFLP type from four different types of loose cooked meats (ham, tongue, beef and chicken) as well as environmental samples from the meat display counter, a chiller and a meat slicer.	(PHE unpublished)

NK, not known; L, London Region; E, East Region; NW, North West Region; Y&H, Yorkshire and Humberside Region; CSF, cerebrospinal fluid; AFLP, amplified fragment polymorphism; fAFLP, fluorescent amplified fragment polymorphism; PFGE, pulsed field gel electrophoresis; cfu, colony-forming units.

#### Outbreaks in the community

Amongst all eight outbreaks (designated O1–O8), five of these have been previously described [11, 14, 19, 20]. For all the outbreaks, the implicated food types were all ready-to-eat products of animal origin (Table 4). There was one large outbreak of 378 cases (O1, associated with consumption of pâté), four outbreaks with 10–17 cases (O2, O3, O4 and O5) and the remaining three outbreaks comprised between three and five cases (O6, O7 and O8). Five of the outbreaks occurred over periods of between 1 and 7 months (O2, O3, O4, O7 and O8) and the remaining three (O1, O5 and O6) over several years. Cases were confined to one or two regions for five of the outbreaks (O2, O5, O6, O7 and O8), with the remaining three (O1, O3 and O4) occurring across five or more regions including two outbreaks (O1 and O3) with cases in Scotland and or Northern Ireland.

In all eight outbreaks, *L. monocytogenes* was shown to be indistinguishable between patient's clinical specimens and food samples and there was strong strength of evidence linking consumption of the specific foods to listeriosis both on epidemiological and microbiological grounds. Analytical epidemiology showed significant associations in four of the outbreaks (O1, O2, O3 and O5), with the remaining four having descriptive evidence only. *L. monocytogenes* strains associated with the cases were recovered from foods collected from: a patient's domestic refrigerator in one outbreak (O3); the same retailers used by the patients in three outbreaks (O1, O2 and O5) and from foods or environmental sites collected at the point of production in seven outbreaks (O2–8). In outbreak O1, the outbreak strain was recovered by unrelated testing of pâté collected from a domestic refrigerator of an individual who did not have evidence of listeriosis but was being investigated as part of an outbreak of foodborne infection.

In seven of the eight outbreaks (all except O5), levels of *L. monocytogenes* in foods at >10^2^ cfu/g were detected at some point in the food chain, and of these, levels of >10^3^ cfu/g were detected in four outbreaks (O1, O3, O4 and O8). In outbreak O5, levels of <20 cfu/g were detected in products (pork pies) on the retail sale and at the point of production. However, the outbreak strain was detected at 10^4^ cfu/g in a single pork pie from the point of sale but there was no information available on storage between purchase and submission to the laboratory for testing [19].

Specific foods from six outbreaks were initially implicated when isolates recovered as part of unrelated food testing were submitted to the reference laboratory and, following application of typing, were recognised as indistinguishable to isolates from the clusters of clinical cases (Table 3). The unrelated testing occurred during the outbreak in two instances (O1 and O2), and in four outbreaks *L. monocytogenes* was isolated prior to the onset of the first case by 3 months (O7), 9 months (O4), 1.75 years (O8) or 2.5 years (O6). The considerable lengths of time between recognition of hygiene problems and the onset of the first cases in the outbreaks represent missed opportunities for prevention of this disease. The remaining two outbreaks were initially identified either by testing foods from the patient's refrigerator (O3) or by the patient linking consumption of the specific food (pork pies) with infection (O5, see above).

#### Sporadic cases in the community

Amongst the 10 sporadic cases (designated S1–10; Table 5), five have been previously described [7–10, 18]. For all the incidents, the implicated food types were of animal origin in eight: four were cooked sliced meats (S5, S6, S9 and S10), one was cooked chopped liver (S7), one cooked chicken (S3) and two soft cheese (S1 and S2). The remaining two incidents were associated with foods of non-animal origin: one with marinated olives (S8) and one with vegetable rennet (S4).

In all 10 sporadic cases, *L. monocytogenes* isolated from the patient's clinical specimens and the implicated food were indistinguishable by typing, and each case had strong epidemiological associations in that there were descriptive linkages with the implicated foods. The implicated strain was recovered from food collected from five of the patients' domestic or institutional refrigerators (S1, S3, S4, S7 and S8) and also subsequently from food sampled at retailers used by two of the patient's (S2 and S7). The implicated strains were detected in food or environmental samples collected at production or distribution for seven of the cases (S2, S5, S6, S7, S8, S9 and S10) and consequently this constituted strong microbiological evidence linking the specific food to the patients' infection: the specific strain was only isolated on one occasion from a product collected at retail for S6. The implicated strain was only recovered from foods collected from patients' domestic refrigerators for three of the cases (S1, S3 and S4) and these three incidents did not have strong microbiological evidence to implicate the specific foods.

Levels of *L. monocytogenes* in foods were not available for three of the sporadic cases (S3, S4 and S10). For the remaining seven, the levels were described as heavy in two (S1 and S2), and at any point in the food chain, were at a maximum of 40 (S8), >10^2^ (S6) and >10^3^ cfu/g (S5, S7 and S9).

The initial evidence linking specific foods to the listeriosis patients was: in five incidents by the collection and testing of foods from the patients' domestic or institutional refrigerator (S1, S3, S4, S7 and S8), in two incidents by testing foods collected in unrelated surveys (S5 and S9) and in the final three cases by testing foods collected from a retailer identified by the patient (S2, S6 and S10; Table 3). In the two instances where unrelated sampling and typing of isolates initially identified a link between the case and the foods, the sampling occurred close to the onset of infection for S5 and 6 months prior to onset for S9.

### Considerations on foodborne listeriosis in the community

The identification of incidents of foodborne illness in the community can be problematic and there are different strengths of evidence implicating suspected foods vehicle. The evidence to attribute a particular food vehicle can be epidemiological, microbiological, descriptive environmental or based on product-tracing investigations [21]. Patients related by common food exposures can be widely separated both geographically and temporally because of the features of listeriosis i.e. the long incubation period, low attack rate, prolonged colonisation at food production facilities and the complexity of the food chain. Strong epidemiological evidence includes statistical associations in well-conducted analytical epidemiological studies or convincing descriptive evidence. For listeriosis, statistical associations may be difficult to apply since outbreaks generally affect relatively small numbers of cases: in this series, all except one of the outbreaks affected 17 or fewer patients. Furthermore, descriptive evidence may be subjective and based on value judgements, e.g. convincing descriptive evidence.

Microbiological evidence includes the detection of the causative agent in the food vehicle or its components and the detection of the causative agent in the food chain or from the preparation or processing environment. For investigating listeriosis this may be problematic, for example, the recovery of indistinguishable *L. monocytogenes* from the patient's clinical specimens and from food which had been in direct contact with the patient (such as from their domestic refrigerator) or from opened food collected in a single setting are not considered to be of a strong association since cross-contamination between foods or from the patient to a food cannot be excluded. The recovery of indistinguishable *L. monocytogenes* from a patient and from food or food components at manufacture is considered as strong microbiological evidence. Since *L. monocytogenes* can persist in environments for considerable lengths of time, environmental studies may be inconclusive in providing evidence for specific exposures, particularly if there is cross-contamination within a complex food chain. Product-tracing (investigating the movement of a food product and its constituents through the stages of production, processing and distribution) may be difficult, complex and time consuming. Advances in the characterisation of *L. monocytogenes* isolated from different parts of the food chain may allow targeting of this process. Consequently, for the investigation of human listeriosis, no one data source is likely to be sufficient for public health investigations and epidemiological and microbiological evidence for the infection should be combined with data on the causative agent in the food chain. Furthermore, because of the ability of *L. monocytogenes* to persist in food environments for years to decades [3], it is also important to be able to integrate these data over many years on a national or even an international basis, and interpret these on the background of the evolutionary changes of the bacterium.

Amongst all the cases in the community, there were 10 sporadic cases and eight outbreaks identified as associated with foodborne transmission. The majority of cases were part of outbreaks comprised <14 cases and continued for over 1 month to 3 years. Descriptive studies were used for all outbreaks and analytical epidemiology was successfully applied to half of these. The large outbreak associated with pâté consumption [11] occurred over all regions in the UK and reflects the national distribution of this product. Half of the outbreaks in the community were confined to a single region, and eight of the nine nosocomial outbreaks were confined to a single hospital: this is likely to reflect more local food producers with restricted (although sometimes rather complex) distribution chains.

Because of the difficulties in attributing specific foods to the transmission of listeriosis, it is important to combine multiple sources of data. Including data from sporadic cases with that from outbreaks is likely to provide additional information on risk factors including those foods of greatest risk. Using all the data here, the most common information source to initially identify the specific foods associated with the cases was typing of isolates from food or the environment either recovered as part of unrelated food testing, or from food items recovered from patient's domestic refrigerators. This may be surprising since there is a very low probability of sampling foods associated with transmission of disease at random and independently of investigations of cases of suspect foodborne listeriosis. However, sampling is not carried out at random. PHE currently manages a network of Official Control laboratories in England which test approximately 25,000 food and environmental samples for the presence of *Listeria* each year. This activity generates more than 700 *L. monocytogenes* isolates being sent for characterisation to the reference laboratory. Cultures are also received by the reference laboratory which were isolated by Official Control laboratories in Wales, as well as from commercial food microbiology laboratories. There is often a risk basis for the collection of samples, either because of direct observations of hygiene practices (samples are more likely to be collected where hygiene is poor) as well as from prior knowledge of foods previously associated with transmission of listeriosis. Consequently, the analysis of *L. monocytogenes* (as well as for other enteric pathogens) isolated from food and the environment provides a unique source of data for surveillance. With the advent of the more widespread application of WGS, there is an urgent need to consolidate both national and international repositories of data such that public health investigations, including outbreak and incident investigation, risk assessment, as well as strategies for disease prevention and control can be rapidly identified and interventions in the food chain implemented [39].

Following the typing of isolates from unrelated food testing, the second most common method of initially identifying incriminated foods in incidents of listeriosis was through testing items recovered from patient's domestic refrigerators. The advantage of testing foods collected from patients' homes was demonstrated in an outbreak of 34 listeriosis cases which occurred in Canada in 2016 [40]. In this Canadian outbreak, the incriminated food was not identified by a case control study, but following the recovery of the implicated strain from pasteurised chocolate milk collected from one of the patient's homes. After re-interviewing 12 of the patients, seven identified exposure to this product, and this led to the sampling and recovery of the implicated strain from the food production site. Although foods may no longer be available in domestic refrigerators since listeriosis patients show variable (1–90 days) incubation period [4, 5], the conservative nature of an individuals' eating habits and the colonisation of harbourage sites in food production environments suggest that collection of samples from domestic refrigerators (or freezers) may be valuable. In the light of the generally low attribution of specific foods to patients with listeriosis, we would advocate both better linkage of data from unrelated testing of isolates as well as investigation of foods from patients' refrigerators as part of the routine investigation of listeriosis cases. The importance of contamination of foods from harbourage sites within food production facilities has long been recognised [2]. Incidents of listeriosis are almost always associated with this route of contamination for ready-to-eat foods where this bacterium may subsequently grow during storage and prior to consumption [2]. Considerations on the levels of *L. monocytogenes* contamination in food and the environment together with the likelihood of growth and survival of the bacterium are important to consider since risk will increase with the level of exposure. Data from the series described here implicated foods which were capable of supporting growth of this bacterium and were processed. The most common types of food contained ingredients of animal origin (cooked chicken, pâté, pork pies, sliced cooked meats, sliced ox tongue, crab meat, butter and soft cheese), although foods of non-animal origin (marinated olives and vegetable rennet) also occurred in this series. The foods described here are generally typical of those described in other countries [2] and are ready to eat and generally able to support the growth of this bacterium. The more ‘unusual’ food vehicles in this series were butter and olives. Butter has previously been implicated in an outbreak of listeriosis in Finland [41]. The Finnish outbreak was associated with 7 g individual packages whereas in the outbreak described here, 2 kg packs were implicated (Table 4). In the English outbreak reported here there was anecdotal information that microwave heating was being used to soften the butter allowing easier spreading onto sandwiches. The detection of the implicated *L. monocytogenes* strain at 1.8 × 10^2^ cfu/g in butter suggests that either there is gross contamination of the milk at the start of the process (this strain was present in the drain within the dairy) or that growth was occurring in the butter matrix which might have been degraded by the microwave heating. A study was subsequently carried out on butter on retail sale in the UK [42] which demonstrated that although butter is regarded as a low-risk product, it may provide an environment for the persistence and growth of *Listeria* spp., including *L. monocytogenes*. Olives are generally not a permissive environment for the survival and growth of *L. monocytogenes* [43]. In the sporadic case described here (Table 5) the olives were marinated in garlic and sweet chili oil and the implicated *L. monocytogenes* strain was recovered on one occasion at 40 cfu/g suggesting some growth had occurred, particularly at a market setting without refrigeration. Data on the pH (which will limit growth if sufficiently acidic) of this product is not available but this bacterium will grow in high salt environments [2] and there is a case described in Finland associated with consumption of salted mushrooms [44]. The importance of food of non-animal origin in the transmission of listeriosis is increasingly being recognised [34, 45].

For the sporadic cases and outbreaks in the community (unlike those in hospitals), the levels of the specific *L. monocytogenes* strains in food were generally high (>10^2^ cfu/g) at some point in the food chain, and indicates that the specific foods supported the growth of this bacterium. In data generated by both PHE from routine testing of foods and across the EU [1], this level is rare and exceeds limits in statutory microbiological criteria [46]. In some instances, these levels were detected several years before the first cases of listeriosis were detected. Food manufacturers and food regulators should make stringent efforts to ensure that foods with elevated high levels of *L. monocytogenes* do not occur in the food chain.

In summary, we review here a total of 28 incidents (480 cases) of foodborne listeriosis in England and Wales between 1981 and 2015 where specific foods were identified as associated with transmission. This report highlights that for public health interventions to prevent human listeriosis, there is a need to combine, and have rapid access to, epidemiological and clinical microbiological data together with the occurrence and behaviour of *L. monocytogenes* in food chains and the environment. In addition, there is a need to apply discriminatory typing systems to all isolates of *L. monocytogenes*. *L. monocytogenes* recovered from unrelated testing of samples from the food chain together with the sampling of foods from patient's domestic refrigerators should be considered essential components of surveillance strategies for human listeriosis. It is also important to be able to integrate data over many years on a national as well as an international basis. The recognition between hygiene problems within food chains prior to the onset of infection represents missed opportunities for the prevention of this disease. Finally, food manufacturers and food regulators should make more stringent efforts to ensure that foods with elevated high levels of *L. monocytogenes* do not occur in the food chain, although greater stringency should be applied to the presence of this bacterium for foods served in hospitals as well as other healthcare institutions which serve food to the elderly and immunocompromised.
